# Central Apneic Event Prevalence in REM and NREM Sleep in OSA Patients: A Retrospective, Exploratory Study

**DOI:** 10.3390/biology12020298

**Published:** 2023-02-14

**Authors:** Katharina Ludwig, Sebastian Malatantis-Ewert, Tilman Huppertz, Katharina Bahr-Hamm, Christopher Seifen, Johannes Pordzik, Christoph Matthias, Perikles Simon, Haralampos Gouveris

**Affiliations:** 1 Sleep Medicine Center, Department of Otorhinolaryngology, University Medical Center, Johannes Gutenberg-University Mainz, 55131 Mainz, Germany; 2 Department of Sport Medicine, Rehabilitation and Disease Prevention, Faculty of Social Science, Media and Sport, Johannes Gutenberg-University Mainz, 55131 Mainz, Germany

**Keywords:** central sleep apnea, sleep stages, polysomnography, chemosensitivity, obstructive sleep apnea, REM sleep

## Abstract

**Simple Summary:**

Obstructive sleep apnea is the most common breathing-related sleep disorder. In addition to the quantitatively dominant obstructive apneas, patients may also be affected by central apneas. This study investigates the frequency of occurrence of central apneas in REM and NREM sleep in patients suffering from OSA of varying severity. When adjusted for the respective REM and NREM sleep duration, a significantly increased frequency of CAEs in NREM was found only in severely affected OSA patients.

**Abstract:**

Patients with sleep-disordered breathing show a combination of different respiratory events (central, obstructive, mixed), with one type being predominant. We observed a reduced prevalence of central apneic events (CAEs) during REM sleep compared to NREM sleep in patients with predominant obstructive sleep apnea (OSA). The aim of this retrospective, exploratory study was to describe this finding and to suggest pathophysiological explanations. The polysomnography (PSG) data of 141 OSA patients were assessed for the prevalence of CAEs during REM and NREM sleep. On the basis of the apnea–hypopnea index (AHI), patients were divided into three OSA severity groups (mild: AHI < 15/h; moderate: AHI = 15–30/h; severe: AHI > 30/h). We compared the frequency of CAEs adjusted for the relative length of REM and NREM sleep time, and a significantly increased frequency of CAEs in NREM was found only in severely affected OSA patients. Given that the emergence of CAEs is strongly associated with the chemosensitivity of the brainstem nuclei regulating breathing mechanics in humans, a sleep-stage-dependent chemosensitivity is proposed. REM-sleep-associated neuronal circuits in humans may act protectively against the emergence of CAEs, possibly by reducing chemosensitivity. On the contrary, a significant increase in the chemosensitivity of the brainstem nuclei during NREM sleep is suggested.

## 1. Introduction

Obstructive sleep apnea (OSA) is a common disorder in the general population. It may be present both in patients without other pre-existing conditions as well as in patients with comorbidities, e.g., heart failure. OSA is associated with increased cardiovascular risk [1,2] The disease itself has a strong negative effect on the health, alertness, and productivity of patients [3]. In a large population-based study (“HypnoLaus study”) in central Europe, the prevalence of moderate-to-severe sleep-disordered breathing in the adult general population was quite high, being 23.4% in women and 49.7% in men [4]. The severity of the disease is associated with a variety of individual risk factors, such as age, body mass index (BMI), smoking, and alcohol consumption [5]. Obstructive sleep apnea is diagnosed in the clinical routine using polysomnography (PSG) or home sleep testing (HST). In this process, obstructive sleep apnea is diagnosed when the breathing disorder cannot be explained by any other sleep disorder, medical condition, medication, or other substance. In addition, an apnea–hypopnea index (AHI) ≥ 15/h (each event ≥ 10 s) sleep time or an AHI ≥ 5/h sleep time in combination with typical clinical symptoms or relevant comorbidity must be present [6]. The pathogenesis of OSA is based on complex central nervous and/or neuromuscular processes that lead to changes in central respiratory regulation and/or upper airway muscle tone during sleep [7,8,9]. In this context, it is known that the severity of OSA between sleep stages, within an individual, can vary considerably [10,11]. Some studies have tried to elucidate the mechanisms underlying the dependence of OSA severity on sleep stages. The focus has been on the main pathophysiological mechanisms of the disease. These include upper airway collapsibility, muscle responsiveness, arousal threshold, and ventilatory control stability (loop gain) [7,8]. Loop gain is defined as the corrective respiratory response divided by the magnitude of the respiratory disturbance that produced the correction. A large respiratory response to a small perturbation corresponds to a high loop gain and significantly represents unstable respiratory control. One pathophysiological trigger of a high loop gain is excessive chemosensitivity to CO_2_ above and below the level of eupnea [12]. However, the data available to date are very limited in terms of allowing any firm conclusions to be drawn [13,14,15,16,17]. It has been shown that the severity of SDB, in terms of increased AHI, increases in sleep stage N1/N2 compared to N3 (slow wave sleep) [18]. A total of 55% of OSA patients have a prominent increase in the obstructive apneic events (OAEs) during NREM sleep stages compared to REM. However, also, a greater AHI in REM compared to NREM can be a part of the clinical spectrum of OSA, which is called REM-related OSA [19,20]. In mild-to-moderate OSA, there is a wide heterogeneity in the patterns of disease, and thus OSA has been categorized into discrete disease phenotypes such as supine-predominant OSA, REM-predominant OSA, NREM-predominant OSA, or intermittent OSA [21].

In patients with central sleep apnea (a sleep disorder with high loop gain), the breathing disorder often becomes evident in NREM sleep [22]. Similar to our observations in the present study, it has been previously suggested that central apneas may be completely absent in REM sleep [23]. Central sleep apnea is described as a break in airflow during sleep, without associated respiratory effort. Often central sleep apnea occurs as a cyclic alternation of periods of hyperventilation, with periods of apnea, known as Cheyne–Stokes breathing. A further criterion for the diagnosis of central apneic events (CAEs) is a duration of 10 s or more on polysomnography. In clinical practice, patients show not only features of one breathing disorder, but a mixture of obstructive sleep apnea and central apneas. The proportion of the two features may change during the night. For this reason, one often speaks of an overlap of obstructive sleep apnea with central sleep apnea [22]. In our exploratory observation, we found a relatively large cohort of patients who had predominant OSA and showed a complete absence of CAEs in the REM sleep phase. Additionally, when adjusted for the respective REM sleep duration, an increased number of CAEs in NREM was found only in the group of patients severely affected by OSA. The aim of the study was to describe and compare the phenomenon of the frequency of central apneic events occurring in patients suffering from OSA in different degrees of severity in REM and NREM sleep, taking into account the duration of the sleep phases.

## 2. Materials and Methods

In this exploratory study, we used a sample size of 141 patients who underwent inpatient polysomnography for the initial diagnosis of obstructive sleep apnea. Included in the recording, according to current AASM (American Academy of Sleep Medicine, Inc., Darien, IL, USA) standards, were a sleep electroencephalography (EEG), electrooculogram (EOG), electromyography (EMG), electrocardiography (ECG), respiratory flow, snoring, respiratory effort (measured by respiratory impedance plethysmography, by using thoracic and abdominal belts), oxygen saturation, body position, and a video recording during sleep. Nasal airflow was detected by measurement of impact pressure through a nasal sensor that determined pressure fluctuations of the breathed air stream. Thoracic and abdominal excursions, oxyhemoglobin saturation (pulse oxymeter), and body position were simultaneously recorded. Snoring was recorded with a pre-laryngeally fixed microphone. The sampling frequency of the EEG data was 200 Hz. The data were high pass filtered at 0.1 Hz and were not resampled before the analyses. The polysomnographic recordings were performed using the Alice-LE-Diagnostic Sleep System (Philips Healthcare/Respironics, Best, the Netherlands).

All patients had presented to our outpatient department in the Sleep Medicine Center of a tertiary University Medical Center with complaints of snoring and or breathing cessation during sleep reported by their partner or peers. Additionally, all patients had previously undergone an ambulatory HSAT (home sleep apnea testing) by a primary physician that showed an AHI > 5/h. As a consequence, each patient’s PSG was recorded over two nights to circumvent the first night effect and only the data from the second night were used for further analysis [24]. The PSG data were evaluated and visually scored by sleep-medicine-board-certified specialists according to current AASM guidelines [13]. Nasal airflow amplitude reduction greater than 90%, lasting for at least 10 s, was defined as apnea. Hypopnea was defined as an airflow reduction between 50 and 90% with an associated 3% reduction of the blood oxygen saturation (SpO2). Apnea events were further classified into obstructive, central, or mixed on the basis of simultaneous evaluation of nasal airflow and thoracic and abdominal excursion. Physiological EEG arousals (e.g., the one associated with changes in sleep stage) and motor-related arousals were excluded in this study. Each patient underwent a clinical examination prior to polysomnography in accordance with the criteria of the AASM. The clinical examination was performed by trained (specialist) physicians who were not identical to the experts who selected the patient data for the study. To determine the degree of excessive daytime sleepiness, the Epworth Sleepiness Scale (ESS), a self-completion test, was used [25]. Inclusion and exclusion criteria were carefully considered for the patient. Inclusion criterion for data and analysis selection was the diagnosis of OSA. Exclusion criteria were age < 18 years, active malignant tumors (end of definitive oncologic therapy > 5 years), chronic obstructive pulmonary disease (COPD, Gold stages 2–4), Raynaud’s syndrome (due to problems with oxygen saturation measurement), primary CSA, pulmonary hypertension, Arnold–Chiari malformation, sedating medication, severe psychiatric illness, severe insomnia requiring treatment, congestive heart failure (NYHA III or IV), or pre-existing therapy for OSA (e.g., surgery or positive airway pressure therapy), as well as an AHI < 5/h. PSG data from 141 patients suffering from OSA were retrospectively analyzed for the occurrence of central apneas during both REM and NREM sleep. The 141 patients were divided into three groups on the basis of the severity of OSA: mildly affected (AHI 5/h to < 15/h), moderately affected (AHI 15/h to <30/h), severely affected (AHI > 30/h). The collection of data and their analysis are compatible with the principles of the Declaration of Helsinki and approved by the local Institutional Review Board (no. 2018-13942).

As expected, NREM sleep time was much longer than REM sleep time in all subjects. As a result, in the relatively shorter REM sleep duration, even fewer of the already rare central apnea events could be observed. In order to avoid distortions of the data and to evaluate the data conservatively rather than inflating them, we calculated the apnea events in relation to the respective REM sleep duration of each individual. For our calculations, we related the number of central apneic events of each patient to the individual duration of the REM phase (i.e., not to the average in the group) and thus considered each patient individually. Important in this context is that we compared the number of central apneic events (CAEs) as absolute events only within one group of patients, i.e., we compared the number of CAES only within the group of mildly (or moderately or severely) affected patients between REM and NREM sleep. Thereby, we always considered the total sleep time in REM and NREM. Since the proportions of REM and NREM in TST are known to be very different and thus counting only the CAEs in the respective sleep phases does not lead to comparable results, we normalized the duration of NREM and REM to look at sleep times of equal duration (with respect to REM duration). That means we calculated how many CAEs would have occurred in one (calculated) NREM duration if it corresponded to the REM duration. If this normalization is not performed, a comparison of absolute events is difficult because the share of the NREM phase in the total sleep time is significantly larger than the share of the REM phase in the total sleep time. Therefore, the total REM duration of the patient was taken into account, and the number of CAEs in a normalized NREM duration was calculated according to the following equation:

(1) $$\;{\left(Number\;of\;CAEs\;in\;REM\right)}_{norm}=\left(\frac{Number\;of\;CAEs\;in\;NREM}{Duration\;NREM\;\left[h\right]}\right)\times Duration\;REM\left[h\right]$$

The normalized (time adjusted) events of CAEs in NREM and REM nested in the three main groups were compared using Wilcoxon’s rank sign test. We used a global Kruskal–Wallis test to analyze the overall difference between groups. A significant global test with a *p*-value < 0.05 was followed by a post hoc analysis using the Wilcoxon test comparing the adjusted frequency of NREM and REM events within the three OSA severity groups. Post hoc p-values were corrected for multiple comparisons using the Bonferroni correction. We considered a Bonferroni–Holm-corrected p-value of 0.05 to be significant. For the comparison of the parametric data (Shapiro–Wilk test) that showed equal variance (Bartlett’s test for homogeneity) across groups (Table 1) between the three groups, we used ANOVA (analysis of variance) for global testing and Tukey’s HSD test for post hoc comparison (Table 1), while non-parametric data were analyzed using Wilcoxon’s signed rank sum test followed by post hoc testing by Wilcoxon’s test of the mildly affected group against the other two groups (Table 2).

## 3. Results

Patients in the three severity groups were proven to have similar epidemiological data on average (Table 1 and Table 2). We compared the groups against each other, and it was noticeable that the comparison of age, in contrast to BMI, was shown to be non-significant (Table 1). This observation is not consistent with the prevailing hypothesis that OSA is an age-dependent disease [26,27,28,29]. However, we considered a comparatively small number of cases, which is not suitable to test this hypothesis. In addition, on the basis of the data of the medical records, it turned out that there were no patients with diagnosed heart failure of any degree in any of the three subgroups of our cohort.

The mild severity group included 40 patients, of whom 35% were female. Of the 47 patients suffering from moderate OSA, 42.6% were female, and of the 54 patients with severe OSA, 22.2% were female. On the basis of a thorough analysis of the raw data, it turned out that the vast majority of the patients (73.8% of the total 141 patients) had no CAEs during REM sleep at all. In addition, this phenomenon has been observed in each one of the three groups separately: 67.5% of patients with mild OSA showed no CAEs at all in REM sleep, whereas this phenomenon was more prevalent in moderately (80.9%) and severely affected (74.1%) OSA patients. Of note, the proportional duration of the REM sleep phase in relation to the total sleep time (TST) varied significantly among patients and ranged between 2.2% and 24.9% (mean value = 12.9 ± 6.3%) of TST. 

**Figure 1:**
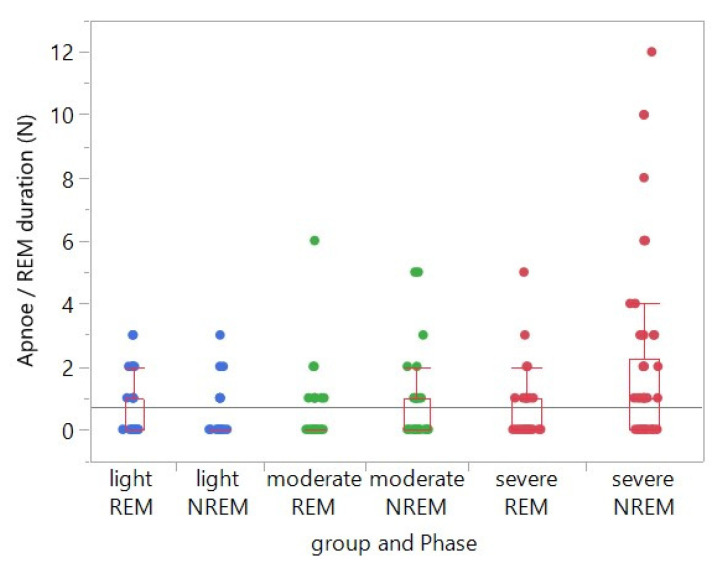
Single factorial analysis of the number of central apneic events (CAEs) in relation to the respective REM sleep duration by group (severity of OSA: mild, moderate, severe) and sleep phase (REM, NREM). In the group of severely affected OSA patients, the frequency of CAEs was significantly increased in the NREM phase when compared to the one in the REM phase, when adjusted for the duration of the REM phase. This was not the case in the group of moderately and mildly affected OSA patients. The symbols overlap in many cases, and most data points were at the value 0. Unfortunately, it is difficult to present this clearly without distorting the intended message of the plot. Due to the overlay, not all data points are clearly visible.

When taking into account this aspect for the data analysis, i.e., if the absolute number of CAEs is related to the duration of the REM phase, then the frequency (number of events divided by time) of CAEs in the REM phase did not differ significantly between the three patient severity groups (mildly, moderately and severely affected) (Figure 1). However, when adjusted for the (individual) duration of the REM sleep phase within the same patient group, the frequency of CAEs in the NREM phase was significantly increased compared to the frequency of CAEs in the REM phase in the group of severely affected patients (*p* = 0.0006). The latter finding was not evident either in the moderately (*p* = 0.1111) or in the mildly (*p* = 0.1158) affected group (Table 3).

In the performed correlation analyses, we were unable to find any correlation of this phenomenon (an increased number of CAEs in NREM sleep in patients with severe OSA) with other parameters (age, body mass index, comorbidities, AHI, ESS score) within one severity group.

## 4. Discussion

In a cohort of 141 patients with OSA, the vast majority did not exhibit central apneic events (CAEs) at all during REM sleep. This observation applies to mildly, moderately, and severely affected sleep apnea patients. In addition, when adjusted for the proportional sleep duration in REM and NREM sleep, the frequency of CAEs was significantly higher in NREM sleep than in REM sleep only in the group of our severely affected OSA patients. This difference was not found in the mildly and moderately affected patients.

The study benefits from the use of a relatively large patient cohort. Of note, the groups of moderately and severely affected patients were very homogeneous regarding the distribution of epidemiological data. In contrast, the group of mildly affected patients presented a lower mean age and mean BMI, an issue that should be considered as a possible confounder. Data from all participants were recorded over two consecutive nights to minimize the first-night effect, and only the data from the second night were used for analysis [24]. The data were all recorded at the same sleep medicine center and were analyzed by a fixed group of experts according to current clinical AASM (American Academy of Sleep Medicine, Inc., Darien, IL, USA) standards. Since this was a retrospective study, the data for ESS, CVRF, or BMI were not available for some patients because they were not recorded at the initial consultation or later documentation (Table 1). Although the study is based on a retrospective data analysis, the data have been very well documented and reviewed. 

To the best of our knowledge, we present in this report the first study describing the phenomenon of completely absent central apneas in REM sleep in a large group (and in a significant proportion) of OSA patients with varying disease severity. Previous studies that look at the associated OSA endotypes (e.g., increased loop gain) in the different sleep stages may have relevance to the occurrence of central events. However, as previously described, most such studies address differences in chemosensitivity between different sleep stages and preferentially include healthy subjects [10]. Reports that are also based on data from OSA patients are of particular interest for the interpretation of our results. We identified one study and one case report that met this condition, a study of 44 OSA patients used the loop gain metric to address the question of how the sensitivity of the ventilatory control system is altered by sleep stage [10,30]; loop gain was lower in REM sleep than in NREM sleep. Since central sleep apnea is associated with a high loop gain, the finding of Landry et al. [10] is, at least in part, consistent with our observation. However, we can confirm these results (when adjusted to the duration of the REM sleep phase) only for the group of severely affected OSA patients, for whom significantly more frequent CAEs could be detected in NREM sleep than in REM sleep. Within the cohorts of the mildly and moderately affected OSA patients, we found no statistically relevant difference between the frequency of CAEs in the REM and NREM sleep.

It should be noted that the studies show that loop gain is not excessively reduced in REM sleep, which is associated with an increase in plant gain [31]. This aspect should be taken into account when considering the differences between OSA severity levels. However, this requires standardized measurement methods and analyses and a large patient population to compensate for variations in physiological measurements [10,31].

Sensitivity of central and peripheral chemoreceptors is an important element in the control of respiratory drive. It has been shown (although mostly in healthy humans) that, as is the case for loop gain itself, chemosensitivity decreases from wakefulness to NREM sleep and is lowest in REM sleep [22,32]. It is assumed that the decrease in chemosensitivity has a protective effect against the occurrence of CAEs. On the other hand, it has been described that it may favor the occurrence of obstructive events (OS) [33], thus explaining the fact that OSA is often more severe during REM sleep. Since we found differences in the frequency of CAEs between REM and NREM sleep only in the group of severely affected OSA patients, one could conclude that a decrease in chemosensitivity and the reduction of loop gain (associated with an increased plant gain) could be responsible for this different frequency of CAEs in REM and NREM sleep.

These statements are in accordance with the observation that central apneas are more frequent in NREM sleep, which has been described before [12,22,34]. Nonetheless, to our knowledge, it has not yet been investigated how the frequency of CAEs relates to the different contributions of REM and NREM sleep to TST in OSA patients. The finding that the frequency of CAEs in relation to the duration of REM sleep differs only in the group of severely affected OSA patients suggests that the pathophysiology of the development of CAEs in OSA patients should be considered differently from the development of CAEs in cohorts of variable OSA severity and of course in cohorts of healthy individuals or cohorts of patients with central sleep apnea.

The Pre-Boetzinger complex (PreBoetC), being one of the four cell groups of the ventral respiratory group (VRG), is the main site of neuronal rhythmic respiratory pattern generation [35,36,37]. In animal models, cells of the PreBoetC were selectively destroyed, resulting in central apneas during sleep [38]. These CAEs occurred initially in REM sleep and, after some time, also in NREM sleep and during wakefulness. The authors concluded that the age-related increase in prevalence of sleep-related breathing disorders may indicate a neurodegenerative background of these disorders. In addition, other authors have found a correlation between age and the frequency of CAEs and offer explanations for it [39]. However, within each OSA severity group, we did not find any significant correlation between the age of the patients and the occurrence or persistence of central apneic events. This means that within a group, i.e., within the group of severely affected OSA patients, no correlation between the age of the individual patient and the prevalence of central apneic events could be proven. Thus, the statistical analysis was performed within a patient group and not between patient groups. For in terms of AHI, obstructive sleep apnea is undoubtedly a disease associated with the aging process and in our data, we were able to find differences in the mean age of the three groups (mildly, moderately, and severely affected) that support this relationship. 

From another perspective, REM-sleep-associated neuronal circuits beyond the brainstem that are connected to the PreBoetC or other VRG sites in humans may act protectively against the emergence of CAEs during REM. Given that emergence of CAEs is strongly associated with the chemosensitivity of the brainstem nuclei regulating breathing mechanics, our results may provide insight into the relationship between decreased chemosensitivity of brainstem nuclei and the severity of OSA [22,37].

The adjustment of CAE frequency to the duration of the REM sleep phase, as performed in the present report, should be considered critically: CAEs are in general rare and occur in very low frequency during REM sleep. In order to compare the absolute values of CAEs in REM and NREM sleep (with their different contributions to TST), the duration of REM sleep was chosen as a reference because it allows for a better depiction of the original data, i.e., the original number of CAEs occurring in REM sleep. The duration of NREM sleep (and thus the number of CAEs) is mathematically reduced with the normalization performed, resulting in lower values of CAEs in NREM sleep. Alternatively, it would have been possible to normalize the duration of REM sleep to the duration of NREM sleep or TST, but this would have led to very high numbers of CAEs in REM sleep, which we do not consider physiologically realistic. Therefore, the observation of rarely occurring CAEs is statistically very ambitious and we hence recommend for future studies on this topic to use even greater patient cohorts than the present one. Likewise, we point out that the analysis regarding the number of central apneic events in REM and NREM was not performed between the groups but only within one patient group. We chose this procedure because it is clear from the sleep parameters that the groups cannot be compared with each other, especially when regarding the comparison of a rarely occurring phenomenon (namely, central apneic events) in relatively short parts of the TST (i.e., REM sleep). The REM sleep duration is so short that it is physiologically impossible to detect a sufficient number of CAEs in one or two PSG nights to be able to calculate meaningful statistics, which we consider as the main limitation of our study. On the basis of our data, we predict that it would take more than five PSG nights to be able to collect sufficient data points. This fact should also be considered for all previously published work in the field to date, but also for future studies. The feasibility of such an approach is, of course, questionable. Nevertheless, we would like to explicitly showcase this quite relevant problem. In addition, these data sets should be used to calculate the loop gain at the different sleep stages and correlate the results with the observations.

To gain further insight into the relationship between the severity of OSA and the occurrence of central apneas during the different sleep phases, prospective studies including pCO_2_ measurements (capnography) during PSG would be of paramount importance. In addition, a parallel consideration of the patients’ cortical arousal profile may provide additional information, given that cortical arousals not only are strongly associated with the severity of respiratory distress in OSA [35] but may also lead to hypocapnia via arousal-related increased chemosensitivity patterns, which may suppress the respiratory drive in OSA patients [40,41,42,43].

## 5. Conclusions

In summary, we demonstrated, apart from the known fact that central apneas are less frequent during REM sleep than during NREM sleep in OSA patients, that a great majority of OSA patients do not have any central apneas at all during REM. In addition, we provide novel evidence that when adjusted to the respective REM sleep duration, an increased number of CAEs in NREM (compared to REM) sleep is found only in the group of patients severely affected by OSA. This difference could not be found in the mildly and moderately affected patients. The pathophysiological mechanisms are unknown so far, but our findings support a correlation between chemosensitivity and OSA severity. Further studies are needed to investigate the relationship between these parameters in more detail.

## Figures and Tables

**Table 1:** The epidemiological data of the 141 patients divided in the severity groups (mild, moderate, and severe) showing the variables age in years, BMI in kg/m², ESS (Epworth Sleepiness Scale) on a scale from 0 to 24, TST (total sleep time) in minutes, TST in REM as a percentage, the proportion of sleep time in supine position of TST as a percentage, and the sleep efficiency as a percentage. The CVRF value stands for the number of cardiovascular risk factors. These were counted according to the patient’s health history (e.g., hypertension, obesity, hyperlipoproteinemia, diabetes mellitus). If the patient has two risk factors, the CVRF value is equal to 2. Besides the grouping, the number of patients, the mean value, the standard deviation, and the upper/lower 95% confidence interval are shown. To test for significance, the analysis of variance (ANOVA) was used together with the Tukey HSD test. We compared the moderately and severely affected groups against the mildly affected group. n.s.: not significant.

Variable	Severity Group	Number	Mean Value	Standard Deviation	95% Confidence Interval Lower Limit	95% Confidence Interval Upper Limit	Global Testing (ANOVA)	Post Hoc (All Pairs Tukey) against “Mild”
Age (years)	mild	40	46.25	14.58	41.57	50.92	n.s.	
	moderate	47	50.54	11.25	47.25	53.86	n.s.	
	severe	55	52.75	13.04	49.22	56.26	n.s.	
BMI (kg/m^2^)	mild	36	26.54	4.69	24.96	28.15	<0.0001	
	moderate	47	30.99	5.60	29.35	32.62	<0.0001	0.0005
	severe	55	31.69	5.11	30.32	33.09	<0.0001	<0.0001
ESS	mild	14	9.13	4.35	6.62	11.64	n.s.	
	moderate	45	9.8	4.76	8.37	11.22	n.s.	
	severe	54	10.15	5.17	8.72	11.55	n.s.	
CVRF (count)	mild	40	0.93	0.93	0.61	1.23	0.0005	
	moderate	47	1.22	0.87	0.97	1.49	0.0005	0.0274
	severe	53	1.76	1.22	1.44	2.11	0.0005	0.0004
TST (min)	mild	40	362.09	55.54	344.31	379.84	0.0483	
	moderate	47	362.33	54.21	346.39	378.25	0.0483	n.s.
	severe	55	336.84	65.37	319.18	354.52	0.0483	n.s.
REM in TST (%)	mild	40	15.32	5.77	13.46	17.15	0.001	
	moderate	47	13.36	6.17	11.53	15.16	0.001	n.s.
	severe	55	10.62	6.11	8.97	12.27	0.001	0.0008
Supine Pos. in TST (%)	mild	40	32.35	25.44	24.19	40.49	0.0213	
	moderate	47	46.47	27.48	38.39	54.55	0.0213	n.s.
	severe	55	47.56	30.32	39.36	55.74	0.0213	0.0273
Sleep efficiency (%)	mild	40	77.38	11.62	73.68	81.11	0.003	
	moderate	47	85.76	8.57	83.24	88.28	0.003	
	severe	55	81.52	12.77	78.08	84.97	0.003	0.0002

**Table 2:** The epidemiological data of the 141 patients divided in the severity groups (mild, moderate, and severe) showing the variables AHI in number per hour, Respiratory Disturbance Index (RDI) in number per hour, duration of SpO2 < 90% as a percentage of TST, mean SpO2 in NREM as a percentage, and mean SpO2 in REM as a percentage. Besides the grouping, the median with 25/75 percentile is shown. To test for significance, the Wilcoxon rank-sum test as well as the post hoc Wilcoxon test was used. We compared the moderately and severely affected groups against the mildly affected group.

Variable	Severity Group	Median	25th Percentile	75th Percentile	Global Testing Wilcoxon Rank Sum	Post hoc Wilcoxon against “Mild”
AHI (n/h)	mild	6.2	4.1	10.7	0.0001	
	moderate	19.5	17.6	23.9	0.0001	0.0001
	severe	48.6	34.9	60.9	0.0001	0.0001
RDI (n/h)	mild	7.5	4.5	11.5	0.0001	
	moderate	20.8	17.6	24.4	0.0001	0.0001
	severe	48.6	36.1	60.9	0.0001	0.0001
Duration < 90% SpO2 (%)	mild	0.08	0.00	0.74	0.0001	
	moderate	0.78	0.15	2.44	0.0001	0.0004
	severe	3.74	0.74	13.08	0.0001	0.0001
Mean SpO2 in % NREM (%)	mild	95	94	96	0.0001	
	moderate	94	93	95	0.0001	0.0043
	severe	93	92	94	0.0001	0.0001
Mean SpO2 in % NREM (%)	mild	95	93	96	0.0001	
	moderate	94	93	96	0.0001	0.0010
	severe	93	91	95	0.0001	0.0001

**Table 3:** Single factorial analysis of the number of central apneic events (CAEs) in relation to the respective REM sleep duration by group (severity of OSA: mild, moderate, severe) and sleep phase (REM, NREM).

Category 1	Category 2	Mean Score Difference	Standard Error Difference	Z-Score	*p*-Value
Severe NREM	Severe REM	18.1418	5.306820	3.41978	0.0006
Moderate NREM	Moderate REM	6.9362	4.353624	1.59320	0.1111
Mild NREM	Mild REM	−6.2000	3.942024	−1.57280	0.1158

## Data Availability

The data that support the findings of this study are available on reasonable request from the corresponding author. The data are not publicly available due to restrictions, e.g., their containing information that could compromise the privacy of research participants.
